# Population pharmacokinetic modeling of dexmedetomidine nasal spray in Chinese adults

**DOI:** 10.3389/fphar.2025.1662364

**Published:** 2025-09-02

**Authors:** Yuanyuan Huang, Sheng Xu, Nassim Djebli, Hao Jiang, Guoping Yang

**Affiliations:** ^1^ Xiangya School of Pharmaceutical Sciences, Central South University, Changsha, China; ^2^ Jiangsu Hengrui Pharmaceuticals Co. Ltd., Lianyungang, China; ^3^ Luzsana Biotechnology Inc, Princeton, NJ, United States; ^4^ Center of Clinical Pharmacology, Third Xiangya Hospital, Central South University, Changsha, China

**Keywords:** dexmedetomidine, population pharmacokinetics, nasal spray, NONMEM, model validation

## Abstract

**Objective:**

The main objective was to build and qualify a population pharmacokinetic (PopPK) model for dexmedetomidine nasal spray in Chinese adults and explore the covariates affecting the PopPK model parameters.

**Methods:**

A population pharmacokinetic model was developed based on the results of 1,225 blood concentration points from 196 healthy volunteers (HV) and patients in phase I and phase III studies. Covariates significantly affecting pharmacokinetic characteristics were analyzed. Model selection was performed using nonlinear mixed-effects modeling (NONMEM), and covariates’ screening was conducted using the traditional stepwise forward inclusion and backward elimination methods. Bootstrap and pcVPC methods were used for model validation. Logistic regression modeling was used to analyze the relationship between the C_max_ within 45 min and the proportion of subjects who achieved Ramsay Sedation Scale (RSS)≥3 within 45 min of intranasal administration.

**Results:**

The final model was a two-compartment model with first-order absorption and linear elimination. Inter-individual variability terms were estimated on clearance and absorption rate constant. The residual variability was described using combined proportional and additive error models. In the final model, body weight was included via theory-based allometric scaling (i.e., exponents of 0.75 for clearances and 1.0 for volumes of distribution). The absorption rate in the patients from phase III study was approximately 49% of that in the HV from phase I study. The estimated population typical values for CL, V2, Q, Vp, KA, F1, and ALAG in the final model were 35.3 L/h, 21.5 L, 116 L/h, 86.5 L, 0.523 h^-1^, 0.653, and 0.0592 h, respectively. Bootstrap results confirmed the stability and reliability of the model, while pcVPC demonstrated good model fit. Logistic regression modeling revealed a significant exposure-response relationship between C_max_ within 45 min and the proportion of RSS ≥3. The concentration slope was 0.01, while the intercept was 0.27.

**Conclusion:**

The present analysis successfully established a PopPK model for dexmedetomidine nasal spray in Chinese adults, confirming that body weight influences distribution and clearance. This PopPK model is being further explored to support pediatric dose recommendation.

## Introduction

Preoperative anxiety and tension are prevalent among patients undergoing surgery, with reported incidence rates as high as 48% (Abate et al., 2020). This psychological stress not only exacerbates physiological responses, such as increased heart rate and blood pressure, but also interfere with the smooth implementation of surgical and anesthetic procedures, ultimately affecting postoperative recovery and clinical outcomes (Baagil et al., 2023). Effective preoperative sedation and anxiolysis are therefore critical to improving patient comfort and ensuring the success of medical interventions. Among the available pharmacological options, dexmedetomidine has emerged as a particularly promising agent due to its unique ability to provide sedation without significant respiratory depression, making it an ideal choice for preoperative use.

Dexmedetomidine, a highly selective α2-adrenergic receptor agonist, has emerged as a cornerstone in perioperative care due to its unique pharmacological profile (Carollo et al., 2008). Since its approval by the U.S. Food and Drug Administration (FDA) in 1999, dexmedetomidine has been widely used for sedation, analgesia, and sympatholysis in various clinical settings, including intensive care units (ICUs) and operating rooms (Lee, 2019). Its ability to provide “cooperative sedation”, where patients remain calm and responsive, has made it particularly valuable for preoperative sedation and anxiolysis, especially in patients with underlying cardiovascular conditions or those requiring rapid postoperative recovery (Gertler et al., 2001; Wang K. et al., 2019).

In recent years, the off-label use of intranasal dexmedetomidine has gained traction as a convenient and non-invasive alternative to IV administration (Li et al., 2018; Li et al., 2018). Intranasal delivery offers several advantages, including rapid absorption through the highly vascularized nasal mucosa and improved patient compliance, especially in pediatric populations (Del Pizzo and Callahan, 2014; McClean et al., 2023). Despite these benefits, the off-label use of IV formulations for intranasal administration has raised concerns regarding dosing accuracy, bioavailability, and safety, as IV solutions are not optimized for nasal absorption (Iirola et al., 2011).

Recognizing these limitations, Jiangsu Hengrui Pharmaceuticals developed the intranasal dexmedetomidine spray, which became the world’s first approved such product in China in 2023. This formulation is specifically designed for nasal delivery, ensuring consistent dosing and enhanced bioavailability compared to off-label IV solutions (Kuang et al., 2022). As the first-of-its-kind product, it represents a significant advancement in the field of perioperative sedation and anxiolysis. It has completed multiple clinical studies, demonstrating its safety and efficacy in adult patients (Gao et al., 2024; Kuang et al., 2022). These studies indicate that the nasal spray is characterized by relatively high bioavailability, rapid absorption, and a short elimination half-life, making it effective and convenient for clinical use.

This study aims to establish a population pharmacokinetic (PopPK) model for dexmedetomidine nasal spray using data from these clinical trials conducted in China. By exploring the covariates influencing pharmacokinetic (PK) parameters, we seek to provide a robust framework for optimizing dosing strategies and improving clinical outcomes. Furthermore, the findings from this study will serve as a foundation for extrapolating the use of dexmedetomidine nasal spray to pediatric populations, addressing a critical gap in current clinical practice.

## Methods

### Clinical data

The data set used for the PopPK model building were derived from one phase I study (Kuang et al., 2022) and one phase III study (NCT04383418). The description of each clinical study used for the current analysis is summarized in Table 1. The depth of sedation was assessed using the Ramsay Sedation Scale (RSS) in phase III study (Table 2). RSS data was used in exposure-response (ER) analysis which were measured at 15-, 30-, and 45-min post-dose as well as anytime within 45 min post-dose based on the subject’s sedation status.

**TABLE 1:** Summary of clinical studies.

Phase of study	Dose (sample size)	Population	Administration	PK sampling points
Phase I (CTR20191868) (CTR20171118)	Part 1 20 μg (N = 12) 40 μg (N = 12)	Health volunteers	IV (15 min)	Before administration and at 5, 10, 15, 20, 30, 45 min, 1 h, 1.5 h, 2 h, 3 h, 4 h, 6 h, 8 h, 10 h post-dose
	Part 2 150 μg (N = 12)	Health volunteers	Nasal	Before administration and at 5, 10, 15, 20, 30, 45 min, 1 h, 1.5 h, 2 h, 3 h, 4 h, 6 h, 8 h, 10 h, 12 h, 16 h, and 24 h post-dose
	Part 3 100/20 μg (N = 12)	Health volunteers	Nasal	Before administration and at 5, 10, 15, 20, 30, 45 min, 1 h, 1.5 h, 2 h, 3 h, 4 h, 6 h, 8 h, 10 h, 12 h, 16 h, and 24 h post-dose
Phase III (NCT04383418)	75 μg (N = 80) 100 μg (N = 80)	Subjects undergoing elective abdominal surgery (excluding liver surgery) who require general anesthesia, endotracheal intubation, and mechanical ventilation	Nasal	Point 1:All subjects will have their first blood sample collected within 5 min after the first Ramsay evaluation ≥3 (indicating successful sedation). If a subject does not reach Ramsay ≥3 within 45 min, the first blood sample will be collected within 5 min after the Ramsay evaluation at 45 min post-dose Point 2:Approximately half of the subjects will have their second blood sample collected within ±15 min after the completion of skin suturing at the end of the surgery, while the remaining half will have their second blood sample collected within 8 h (±1 h) after the administration of dexmedetomidine nasal spray

**TABLE 2:** Ramsay sedation scale.

Definition	Score
Anxious and agitated or restless or both	1
Cooperative, oriented, and tranquil	2
Responds to commands only	3
Brisk response to a light glabellar tap or loud auditory stimulus	4
Sluggish response to light glabellar tap or loud auditory stimulus	5
No response to light glabellar tap or loud auditory stimulus	6

All studies were sponsored by Jiangsu Hengrui Medicine Co., Ltd., and were carried out in accordance with all applicable laws, the Declaration of Helsinki, and Chinese Good Clinical Practice after obtaining approval from their respective ethics committees. Informed consent was obtained from each participant after they had been informed about the potential risks and benefits of the study, as well as the nature of the research.

### Blood sample collection and analysis

The intensive blood sampling was conducted in the phase I study, while the phase III study was characterized by sparse blood sampling. The time points for blood sampling are detailed in Table 1. The concentration of dexmedetomidine in plasma was determined using high-performance liquid chromatography-tandem mass spectrometry (HPLC-MS/MS), consistent with the method described in previous study (Kuang et al., 2022).

### Pharamcokinetic modeling

NONMEM (Version 7.5), Pirana and Perl Speaks NONMEM (Version 5.4.0) were used for model building and model simulation. The R software package (Version 4.3.3) was used for ER modeling, plotting, and constructing virtual populations. SAS (Version 9.4) was used for organizing and analyzing datasets and performing statistical analysis.

A PopPK base model for the investigational drug was built, including the structural framework of the base model and the modeling description of inter-individual variability (IIV) and residual variability (RV) of the PK parameters. The model structure was explored with both linear and nonlinear elimination using one-compartment and two-compartment models. The structure of the base model was determined by observing the semi-logarithmic concentration-time curves and evaluating the changes in the minimum objective function value (OFV) obtained from the first-order conditional estimation method with interaction (FOCE-I) in the NONMEM. The inter-individual variability (IIV) of the model was described using an exponential error model (Equation 1) and the residual variability (RV) was evaluated using Equations 2–4.

$$P_{i}=P_{TV}\times \exp\left(\eta_{i}\right)$$ (1)

$$Y_{\text{obs}.\text{ij}}=Y_{\text{pred}.\text{ij}}+\varepsilon_{\text{ij}.1}$$ (2)

$$Y_{\text{obs}.\text{ij}}=Y_{\text{pred}.\text{ij}}\times \left(1+\varepsilon_{ij.1}\right)$$ (3)

$$Y_{\text{obs}.\text{ij}}=Y_{\text{pred}.\text{ij}}\times \left(1+\varepsilon_{ij.1}\right)+\varepsilon_{\text{ij}.2}$$ (4)

The covariates under investigation include state (HV vs. patients), sex, body mass index (BMI), body weight (BW), body surface area (BSA), and age. The correlations among these covariates were assessed using graphical methods, and covariates exhibiting significant correlations (correlation coefficient >0.8) were incorporated into the fixed effects model separately to mitigate multicollinearity. The continuous covariates were described using a linear model (Equation 5) or a power model (Equation 6), Categorical covariates were described using a piecewise model (Equation 7). In the equation, θ is the correction factor for individual parameters, COV is the value of the covariate, and COV_median_ is the median or typical value of the covariate in the general population.

$$P_{i}=P_{TV}\times \left[1+\theta \times \left(COV-{COV}_{median}\right)\right]$$ (5)

$$P_{i}=P_{TV}\times {\left(\frac{COV}{{COV}_{median}}\right)}^{\theta }$$ (6)

$$P_{i}=\left\{\begin{array}{l} P_{TV}\;if\;COV=type1\\ P_{TV}\times \left(1+\theta \right)\;if\;COV=type2 \end{array}\right.$$ (7)

Following the characterization of the base model, scatter plots depicting the relationship between the base model parameters and the covariates were generated to proceed with a visual inspection. The final covariates for further examination were determined based on the trends observed in these scatter plots, considering the physiological relevance of the covariates.

In the covariate examination process, the stepwise covariate modeling (SCM) approach was used. Each covariate was introduced into the model sequentially. A decrease in the objective function value (OFV) exceeding 3.84 (P < 0.05) indicated that the inclusion of the covariate significantly enhanced the model fit, warranting its retention in the full covariate model. Subsequently, covariates were one by one removed from the full covariate model to assess their impact once retained in the full model. An increase in the OFV exceeding 10.83 (P < 0.001) led to the retention of the covariate into the model. Otherwise, the covariate was deleted from the model. The modeling standards are detailed in Supplementary Material.

### Model qualification

Goodness of Fit (GOF) plots were used to evaluate the prediction deviation of the final model, including the following aspects: the population and individual predicted concentrations versus observed concentrations, conditional weighted residual errors (CWRES) versus population predicted concentrations, the distribution and correlation of random effects, and individual fit plots.

The predictive performance and stability of the model were validated using a bootstrap method with 1,000 iterations. The bootstrap parameters were used to assess the estimation precision of the model parameters. Specifically, 1,000 new datasets were generated by resampling the original data with replacement, and the model parameters for each dataset were calculated. The median and 95% confidence interval (CI) of the bootstrap parameters were computed using non-parametric statistical methods, specifically the 2.5 and 97.5 percentiles of the 1,000 results. The estimated parameters from the original data were then compared with the 95% CI from the bootstrap results.

Prediction-corrected visual predictive check (pcVPC) was used to evaluate the prediction reliability of the model. Based on the modeling population, simulations were conducted using the population typical values and random effect parameters from the final model to obtain the 5%, 50%, and 95% percentiles of the simulated population’s plasma concentrations. This simulation process was repeated 1,000 times to obtain the median and the 95% prediction intervals for the upper and lower bounds of the percentiles. The overlap between these intervals and the median and percentile intervals of the observed plasma concentration data was compared.

### Exposure-response analysis

From the phase III study data, the ‘success in reaching the expected effect’ was defined using a ≥3 RSS score within 45 min of intranasal administration. A logistic regression analysis was conducted to examine the correlation between C_max_ and the proportion of subjects attaining successful sedation during the Phase III study. C_max_ from the time of dosing until the first occurrence of a RSS score of ≥3 was calculated using individual empirical Bayes estimated PK parameters from the final model. If the RSS score did not reach 3 within 45 min post-dose, then the predicted maximum concentration up to reaching this level was used instead.

### Model simulation

Forest plots were used to visualize the effects of retained covariates on key exposure metrics, quantifying parameter uncertainty across subpopulations. Specifically, a forest plot illustrating the impact of BW on C_max_ was generated using the final model parameters through simulations (n = 1,000 virtual subjects per BW group:40, 60, 80, and 100 kg), according to the BW range in the modeling dataset. All virtual subjects received a fixed intranasal dose of 100 μg.

For pediatric populations, exposure simulations were conducted based on weight ranges. One thousand virtual pediatric subjects per stratum were generated through stratified random sampling across three weight categories (10–20 kg, 20–30 kg, and 30–50 kg) with corresponding 30 μg, 50 μg, and 75 μg doses.

Exposure profiles were simulated using the final pharmacokinetic model for each weight stratum.

## Result

### Data analysis

The final analysis utilized a dataset including 1,225 plasma concentration points from 196 individuals across Phase I and Phase III studies. The percentage of samples below the lower limit of quantification (BLQ) within this analysis was considered to be low (close to 1%), therefore, the M1 method was used to handle the BLQ data (Beal, 2001). A summary of baseline demographic characteristics is presented in Table 3. The age distribution of the population in the 301 Phase III study is broader compared to the two phase 1 studies with a higher proportion of females. The BW, BMI and BSA distributions are similar across the studies.

**TABLE 3:** Baseline characteristics of subjects and patients included in the PopPK data set.

Covariate	Phase I (N = 48)	Phase III (N = 148)	Total (N = 196)
AGE (years)	22 (18–38)	44 (20–65)	38 (18–65)
BW (kg)	57.65 (45.2–82)	60 (46–97)	60 (45.2–97)
BMI (kg/m^2^)	21.46 (19.18–24.80)	23.3 (18.6–29.9)	22.9 (18.6–29.9)
BSA (m^2^)	1.59 (1.34–2.01)	1.61 (1.32–2.19)	1.6 (1.32–2.19)
GENDER (Male/Female)	24/24	40/108	64/132

Notes: Data are expressed as median (min to max).

### Pharamcokinetic modeling

The PopPK base model for dexmedetomidine is a two-compartment model with first-order absorption and linear elimination. The main parameters include central compartment clearance (CL), central compartment volume of distribution (Vc), inter-compartment clearance (Q), peripheral compartment volume of distribution (Vp), absorption rate constant (KA), bioavailability (F1), and absorption lag time (ALAG). The inter-individual variability (IIV) terms were estimated on CL and KA. The model residuals were described using combined (i.e., proportional and additive) model, with BW incorporated via theory-based allometric scaling on distribution and elimination (i.e., on CL, Vc, Vp, and Q) (Sanghavi et al., 2024).

Due to the multicollinearity between BW, BSA and BMI, BSA and BMI were not considered during the covariates’ screening after introducing BW. The impact of age, sex, and state (HV vs. patients) on KA and CL was examined in conjunction with the correlation plots. Through forward inclusion and backward elimination methods, it was ultimately found that there were differences in Ka between healthy volunteers and patients. The resulting model was considered as the final PopPK model. Detailed results can be found in Table 4.

**TABLE 4:** Parameter estimates and bootstrap results of the final model.

Parameter	Final model		Bootstrap^a^	
	Estimates (RSE %)	95% CI	Median	95% CI
CL, L/h	35.3 (3.3)	32.987–37.613	35.34	32.787–37.689
Vc, L	21.5 (6.3)	18.834–24.166	21.51	19.128–25.007
Q, L/h	116 (5.8)	102.907–129.093	115.58	103.304–129.962
Vp, L	86.5 (3.01)	80.6–92.4	86.42	80.384–92.26
KA, h^-1^	0.523 (9)	0.43–0.616	0.53	0.437–0.629
state on KA	1.05 (27.5)	0.603–0.703	1.06	0.525–1.719
F1, %	0.653 (3.9)	0.049–0.069	0.655	0.592–0.706
ALAG,h	0.0592 (8.6)	0.484–1.616	0.0594	0.047–0.068
Inter-individual				
ω (CL), %	22.4 (10.5)	17.79–27.01	22.4	18.037–27.181
ω (KA), %	92.3 (9.5)	75.09–109.51	91.92	74.804–110.033
Residual error				
σ (Prop), %	27.7 (5.9)	24.51–30.89	27.37	24.224–30.6
σ (Add), pg/mL	5.01 (16.7)	3.37–6.65	4.95	3.276–6.815

^a^ The minimization success rate was 99.1% over 1,000 bootstrap iterations.

### Model qualification

**FIGURE 1:**
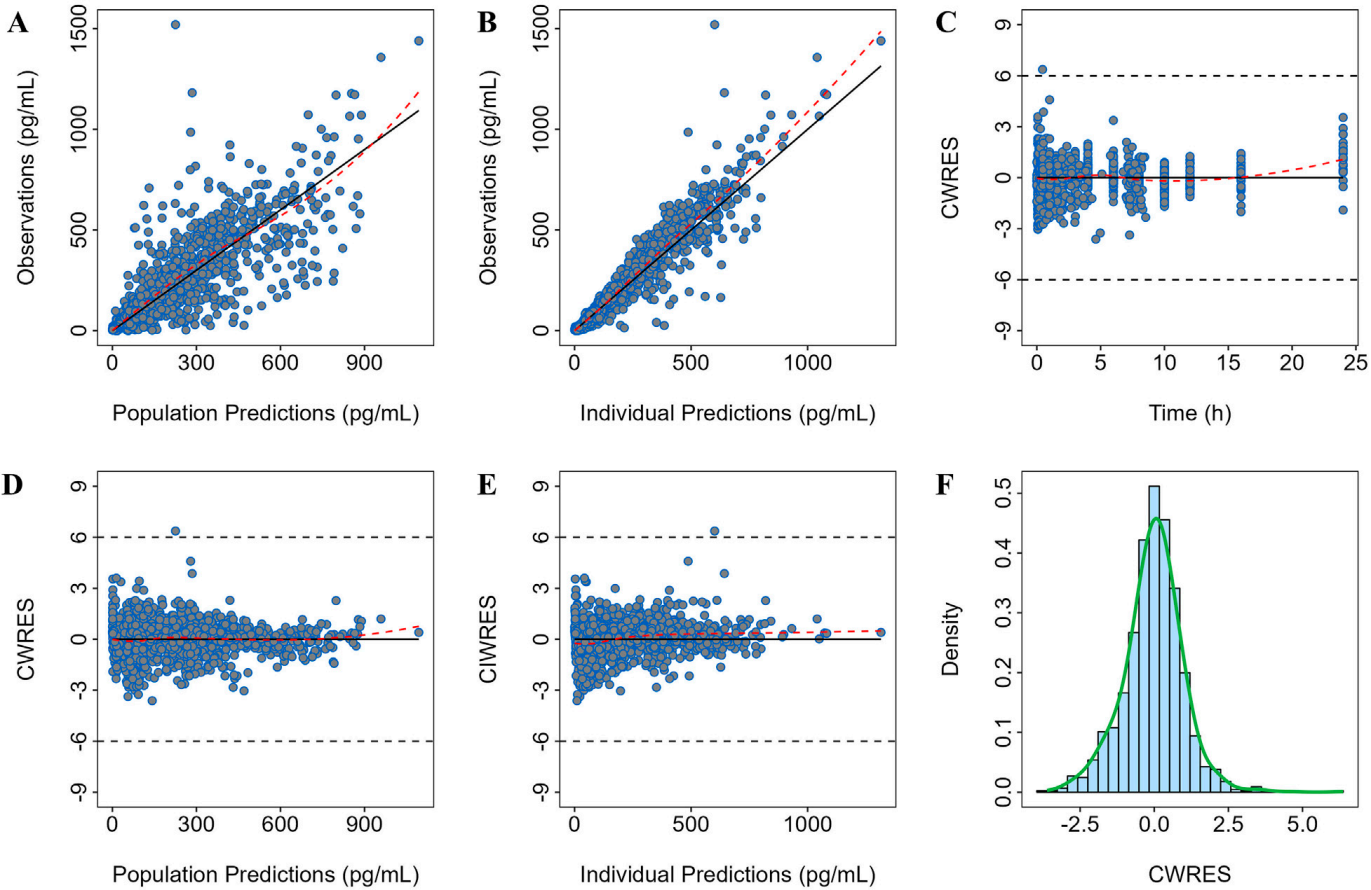
Goodness-of-fit plot of the final model. Observation versus **(A)** population prediction (PRED) and **(B)** individual population prediction (IPRED); **(C)** CWRES versus time; **(D)** CWRES versus PRED and **(E)** IPRED; **(F)** CWRES distribution.

The goodness-of-fit plots for the final model are shown in Figure 1. The regression trend line is close to the standard line, and the conditional weighted residuals (CWRES) values are distributed within ±4, evenly spread above and below the axes.

Internal validation of the model was performed using the bootstrap method, with a success rate of 95.0%, indicating high stability of the PopPK model. The median and 95% confidence intervals (CI) of the bootstrap for the parameters are shown in Table 3. There is a huge overlap between bootstrap and observed median [95% CI], further confirming good precision in parameter estimation.

**FIGURE 2:**
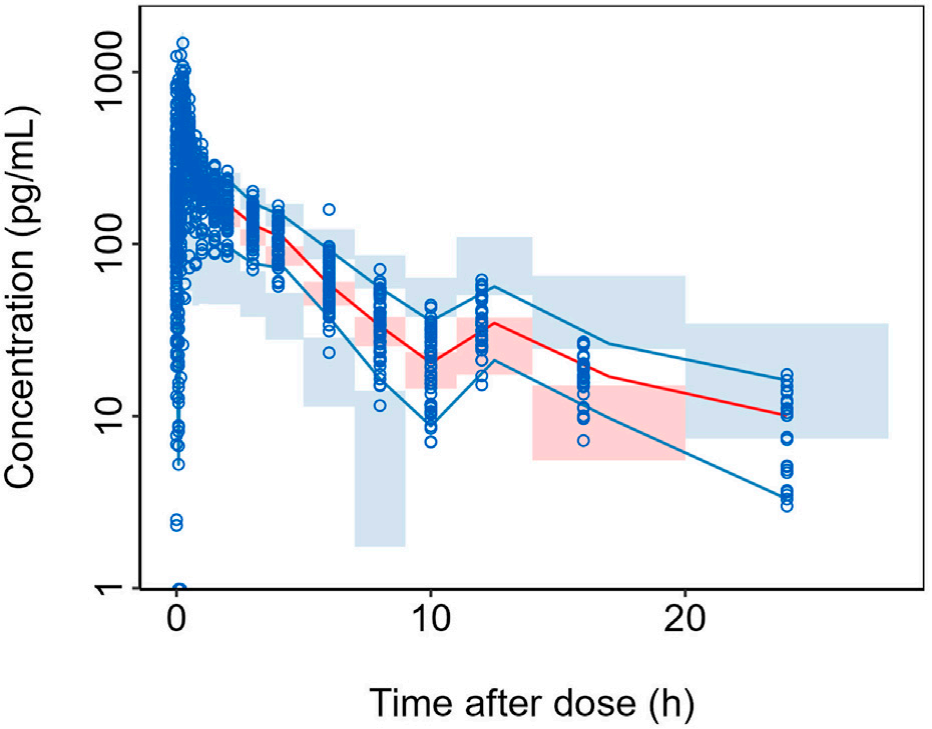
pcVPC of the final model. Semi-logarithmic Scale. For each bin: blue circles represent the observed data points and black solid lines indicate the 5th and 95th percentiles of the observed data; red solid line represents the median of the observed data; blue shaded area shows the 95% CI for the 5th and 95th percentiles as predicted by the model and red shaded area displays the 95% CI for the median as predicted by the model.

The results of the prediction-corrected visual predictive check (pcVPC) are shown in Figure 2, where the median and the upper and lower 5 percentiles of the observed plasma concentrations are overall well captured by the predicted values, and the predictions generally encompass the observed values.

### Exposure-response analysis

**FIGURE 3:**
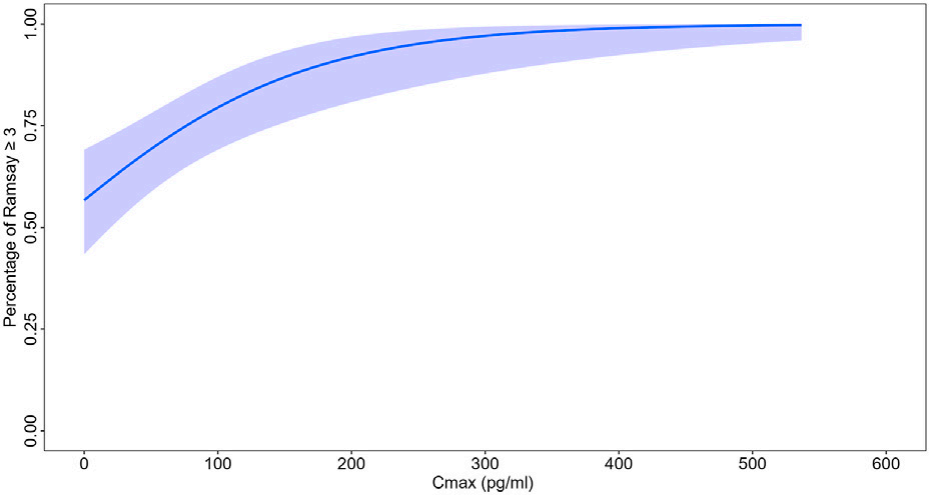
Result of logistic regression.

The logistic regression model shows the significant relationship between C_max_ and the probability of actually achieving RSS score≥3. The intercept was 0.27 and the coefficient for concentration was 0.01 as shown in Figure 3. The p-value associated to Chi-square when performing Anova in R between the null model was <0.001. Specifically, the C_max_ required to achieve 80% and 90% probabilities of attaining RSS score ≥3 was approximately 100 pg/mL and 180 pg/mL, respectively.

### Effects of covariates on exposure

**FIGURE 4:**
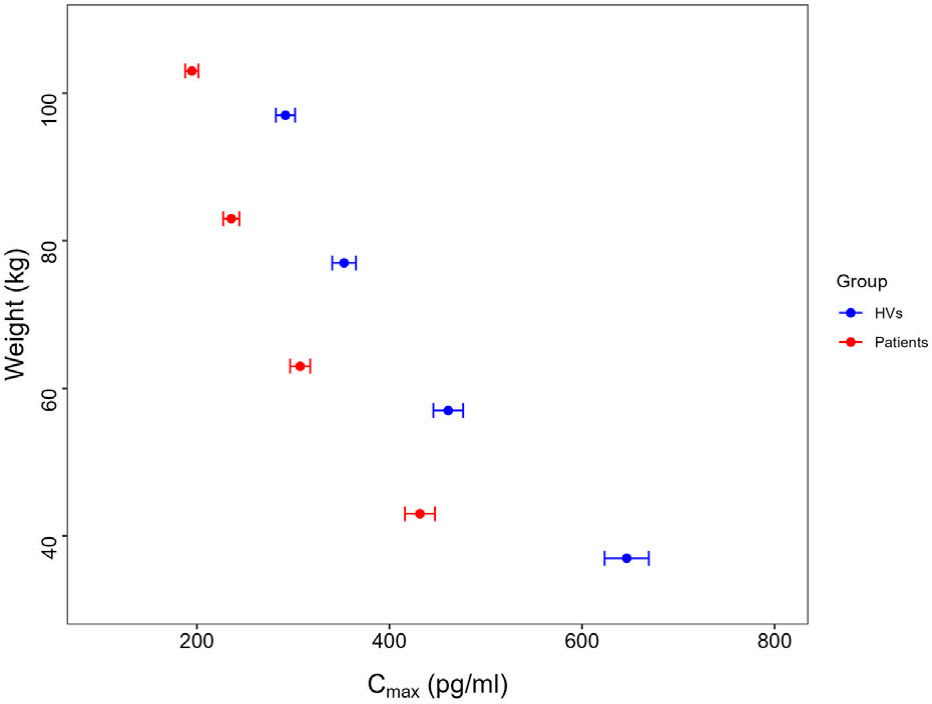
Mean and 95%CI of C_max_.

Given that body weight is a primary covariate influencing drug exposure, and the sedative effects of dexmedetomidine are primarily related to drug concentration (Miller et al., 2018; Potts et al., 2009), the final model was applied for model simulations, assessing the impact of body weight on exposure. The results of simulation are presented in Figure 4. The simulation results showed that as body weight increases, the dexmedetomidine concentration in patients decreases, with the mean C_max_ values for the 40–100 kg population all above 180 pg/mL at a 100 μg dose.

### Pediatric exposure simulation

**FIGURE 5:**
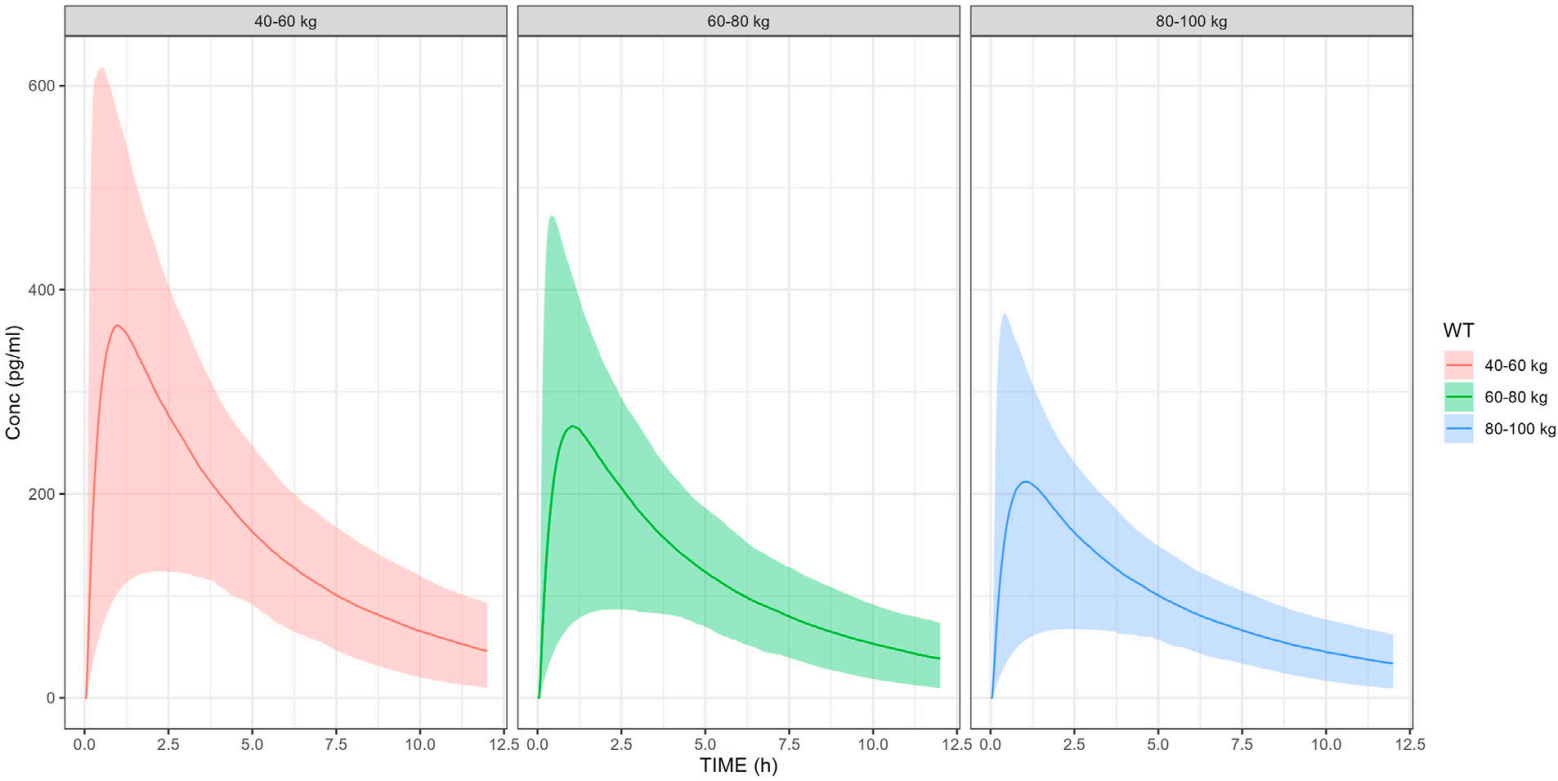
Pediatric exposure simulation by weight-stratified dosing. Solid line: median predicted concentration; shaded area: 90% prediction interval (5th-95th percentiles).

The pediatric exposure simulation results are presented in Figure 5. Following weight-stratified dosing, the mean C_max_ values were approximately 301,317 and 319 pg/mL in the 30 μg (body weight ranging from 10 to 20 kg),50 μg (body weight ranging from 20 to 30 kg) and 75 μg groups (body weight ranging from 30 to 50 kg), respectively. The proportions of subjects achieving C_max_ ≥100 pg/mL were 95.1%,97% and 97.3% respectively in the 30 μg,50 μg and 75 μg groups, while the probabilities of reaching 180 pg/mL were 77.3%,79.5% and 80.9%.

## Discussion

The present analysis describes the PopPK model building and qualification using data from phase I and phase III studies to characterize the PK of dexmedetomidine with IV and nasal spray administration. The plasma concentration profiles were accurately characterized by a two-compartment linear model incorporating a first-order absorption kinetics and a lag time. This model structure has been widely adopted in pharmacokinetic studies of intranasal (IN) dexmedetomidine (Wang C. Y. et al., 2019; Yoo et al., 2015). The KA in healthy volunteers was approximately 1 h^-1^, consistent with previously published analyses (James et al., 2022; Song et al., 2019; Yoo et al., 2015).

The covariates’ screening step revealed significant differences in the KA between healthy volunteers and patients, with the KA in patients being approximately 49% of that of healthy volunteers. The observed difference between healthy volunteers and patients might be partly explained by the distinct blood sampling time point designs in sparse phase III versus rich phase I. The sparsity of sampling points during the absorption phase in patients as compared to rich sampling in healthy volunteers could introduce biases in the estimation of the absorption rate (Ai-Banna et al., 1990; Ogungbenro and Aarons, 2008). Considering the demonstrated efficacy (sedation success rate and onset time) and tolerable safety profile in Phase III study patients, the observed difference in KA might be less pronounced as compared to the estimated value (Gao et al., 2024).

Currently, there are several studies on the use of IV or IN dexmedetomidine in pediatric and infant populations, and PopPK models have been developed based on these studies (Liu et al., 2017; Miller et al., 2018; Song et al., 2019). Furthermore, a previous analysis in which a model was built based with intranasal administration in pediatric populations reported KA of 0.94 h^-1^ which aligns well with the KA value observed in our study of healthy volunteers (Vilo et al., 2008). This consistency is instrumental for validating our approach and supports the reliability of our pharmacokinetic model when applied to pediatric patients.

Body weight was first included as a covariate affecting the volumes of distribution and clearances in the base model via theory-based allometric scaling (i.e., with exponents of 0.75 and 1 for clearances and volumes, respectively). This approach is grounded in physiological principles: the 0.75 exponent for CL aligns with West, Brown, and Enquist (WBE) theory, which describes the allometric relationship between metabolic rate and body mass (West et al., 1997; West et al., 1999), while the exponent of 1.0 for V reflects the direct proportionality between body size and volumes of distribution (Holford and Anderson, 2017). The use of the fixed exponents was further justified by prior pharmacokinetic studies of dexmedetomidine (James et al., 2022; Song et al., 2019),which demonstrated consistency with this scaling method. The simulations across different weight ranges showed that higher body weight is associated with lower exposure to dexmedetomidine. For adults belonging to the highest body weight group (i.e. 100 kg receiving 100 μg), the mean C_max_ was approximately 200 pg/mL. According to the ER analysis, concentrations exceeding 180 pg/mL can achieve a 90% success rate for ideal sedation, which is consistent with the observations from Weerink et al. (Weerink et al., 2017). This indicates that a 100 μg dose is likely to provide stable sedation effect for adults with different body weights, which is also consistent with the results from Phase III clinical trials included in the present analysis. Similarly, it was observed that lower body weight was associated with increased C_max_ values (approximately 416–447 pg/mL). This relatively high C_max_ value remains lower than that observed with the approved injection (∼1,250 pg/mL for 0.70 mcg/kg/hr) (HOSPIRA. PRECEDEX, 2025) indicating that the safety profile is manageable. However, body weights in children are generally smaller, making it necessary to adjust the dose based on their weight to ensure maximizing the therapeutic benefit while mitigating any safety risk. Consequently, a 100 μg dose may not be appropriate for children, as it could lead to higher than desired exposure levels. Stratified dose adjustment based on body weight categories was implemented in pediatric patients, followed by exposure simulation modeling. The results demonstrated comparable exposure profiles between adjusted pediatric doses and adult reference values. Furthermore, the simulation demonstrated ≥95% probability of target attainment (PTA) across all dosing strata for the predefined therapeutic threshold (100 pg/mL) established in adult exposure–response (ER) modeling. These findings suggest that the weight-stratified dosing regimen may provide appropriate therapeutic coverage for the pediatric population. The optimal dose for pediatric use therefore requires further exploration.

Additionally, bioavailability is a particularly critical pharmacokinetic parameter to consider for nasal spray formulations. The final model estimated the bioavailability to be approximately 65%, which is consistent with a previous study conducted in healthy Caucasian adults (Iirola et al., 2011). Nonetheless, we observed that differences in nasal spray formulations and administration procedures increase inter-trial variability (Potts et al., 2009; Vilo et al., 2008). Improper administration procedures may reduce bioavailability and compromise the treatment efficacy (Kuang et al., 2022). This highlights the necessity of standardizing nasal spray specifications and implementing consistent administration protocols. Standardization would help mitigate exposure variability, ensuring uniform safety and efficacy profiles across different studies and patient groups. Specifically, by establishing clear guidelines for formulation composition and administration techniques, we can minimize inconsistencies and improve the overall reliability of intranasal dexmedetomidine therapy.

This study has several limitations. The model did not incorporate hematological and biochemical data for covariate screening due to limited data, which may lead to the omission of additional covariates affecting pharmacokinetic characteristics. Assuming that the primary covariate influencing the PK characteristics of dexmedetomidine is body weight as reported in previous studies, this omission is likely to have a minimal impact. Future studies will expand the model by integrating larger adult and pediatric datasets, along with additional covariates (e.g., genetic factors, comorbidities, and drug-drug interactions), to refine ER relationships for sedation, analgesia, and cardiovascular effects, ultimately validating dosing recommendations across age groups.

## Conclusion

Dexmedetomidine nasal spray improves drug administration convenience and compliance, but demands careful attention to its absorption characteristics. The present analysis describes a PopPK model using data from two studies with healthy volunteers and one Phase III study that includes patients’ to examine the bioavailability and absorption PK characteristics in adults, thereby enhancing its clinical application. The ER analysis showed that a fixed dosage of 100 μg can achieve ideal sedation in adults. This study establishes a theoretical basis for developing a pediatric population PK model, facilitating optimized dosing guidelines and improved clinical outcomes in younger populations. Future work should aim to further characterize the PopPK model with pediatric data.

## Data Availability

The data analyzed in this study is subject to the following licenses/restrictions: This data is confidential proprietary information of the company and cannot be disclosed. Requests to access these datasets should be directed to hao.jiang@hengrui.com.
