# Supplementary material for: Population pharmacokinetic modeling of dexmedetomidine nasal spray in Chinese adults
Source: Front Pharmacol. 2025 Sep 2;16:1662364. doi: 10.3389/fphar.2025.1662364 (PMC12436384; doi:10.3389/fphar.2025.1662364)
Supplement: Supplementary file 1 [file DataSheet1.pdf]

The NONMEM run was verified by meeting the following criteria:

- Minimization obtained successfully in NONMEM.
- Covariance step obtained successfully. In the case in which covariance could not be computed, the standard error was to be estimated using a non-parametric bootstrap.
- At least 3 significant figures should be obtained for all θ estimates. If less than 3 significant figures were obtained for the θ estimates, reasons were to be justified.
- The standard error of θ estimates should preferably be less than 30% and the standard error of Etas estimates should preferably be less than 50% of the estimate itself.
- The shrinkage computed for Eta estimates should preferably be less than 30%.
- There should be no unacceptable trends in the goodness-of-fit plots described in Section Model qualification.
